# 3-D Visualization of Atlantic salmon skin through Ultrasound and Photoacoustic Microscopy

**DOI:** 10.1371/journal.pcbi.1011709

**Published:** 2024-10-22

**Authors:** Abhishek Ranjan, Jaya Kumari Swain, Balpreet Singh Ahluwalia, Frank Melandsø

**Affiliations:** 1 Department of Physics and Technology, UiT, The Arctic University of Norway, Tromsø, Norway; 2 Norwegian College of Fishery Science, Faculty of Biosciences, Fisheries and Economics, UiT, The Arctic University of Norway, Tromsø, Norway; Case Western Reserve University, UNITED STATES OF AMERICA

## Abstract

**Significance:**

Three-dimensional photoacoustic imaging (PAM) has emerged as a promising technique for non-invasive label-free visualization and characterization of biological tissues with high spatial resolution and functional contrast.

**Aim:**

The application of PAM and ultrasound as a microscopy technique of study for Atlantic salmon skin is presented here.

**Approach:**

A custom ultrasound and photoacoustic experimental setup was used for conducting this experiment with a sample preparation method where the salmon skin is embedded in agarose and lifted from the bottom of the petridish.

**Results:**

The results of C-scan, B-scan, and overlayed images of ultrasound and photoacoustic are presented. The results are then analyzed for understanding the pigment map and its relation to salmon behavior to external stimuli. The photoacoustic images are compared with the optical images and analyzed further. A custom colormap and alpha map is designed and the matrices responsible for PAM and ultrasound are inserted together to overlay the ultrasound image and PAM image on top of each other.

**Conclusions:**

In this study, we propose an approach that combines scanning acoustic microscopy (SAM) images with PAM images for providing a comprehensive understanding of the salmon skin tissue. Overlaying acoustic and photoacoustic images enabled unique visualization of tissue morphology, with respect to identification of structural features in the context of their pigment distribution.

## Author summary

Atlantic Salmon is of great importance in Norway for its economic and environmental significance. In this study, Atlantic salmon skin is studied with the aid of ultrasound and photoacoustic microscopy technique. This technique offers several advantages over other microscopy techniques by providing structural, morphological and functional information in detail. We present an overlayed representation of ultrasound and photoacoustic images over each other in three- dimensions. PAM could non-invasively access the multilayered morphological skin structure of salmon fish, allowing for high resolution imaging and analysis without the requirement for labeling. We demonstrated that, when compared to standard optical detection approaches, a visualization strategy based on overlaying fish skin images obtained using SAM and PAM provides a more in-depth view. This method of visualization provides insight into the distribution, variation, and composition of colors inside the live salmon skin without requiring any tissue processing or fixing.

## 1 Introduction

Photoacoustic Microscopy (PAM) is a biomedical imaging modality that detects ultrasound signals generated via optical excitation called the photoacoustic effect [1]enabling deep tissue label-free imaging. PAM is a hybrid imaging modality combining optical excitation and ultrasonic detection allowing a better resolution than traditional ultrasound imaging and achieving deeper penetration depth than purely optical imaging [2]. PAM can generate multi-contrast three-dimensional images ranging from single cells to organoid culture [3]. The abilities of PAM have recently been demonstrated in many applications, including the in-vivo acquisition of valuable anatomical, molecular, functional, and flow dynamic information, towards the elucidation of fundamental mechanisms such as cancer formation and growth [4], the high-resolution monitoring of drug delivery processes [5], as well as the detailed imaging of ocular structures through specialized PAM ophthalmoscopes [6–8].

Traditional imaging methods, such as histology and microscopy, have provided essential information about fish skin anatomy. However, these methods often require invasive procedures, necessitating sample collection, sectioning and potentially disturbing the natural state of the specimen. This is where ultrasound and photoacoustic imaging come to the forefront, offering non-destructive and three-dimensional visualization capabilities. PAM has been applied to many preclinical and clinical skin studies [8–11], for example, Zebrafish have been explored much with PAM and ultrasound earlier. [12]. Photoacoustic tomography has been utilized for high-resolution imaging of zebrafish [13,14]. The whole-body imaging of the zebrafish using multispectral photoacoustic techniques has been performed [15]. Three-dimensional multispectral imaging of human skin have been done for quantifying melanin and blood oxygenation [16]. The randomly non-homogeneous distribution of blood, chromophores and pigments in the skin produces variations in the average optical properties of skin layers.

Despite the evolutionary divergence between aquatic and terrestrial vertebrates, there are similarities in the structural and functional aspects of their integumentary systems. The salmon skin and human skin both have multi- layered structure consisting of epidermis (outer layer) and dermis (inner layer) [17]. In both cases, the epidermis acts as a protective barrier against external threats, while the dermis provides structural support and contains blood vessels, nerves, and other components [18]. These layers work together to provide protection, regulate body temperature, and perform other vital functions. The primary function of skin are quite similar in both the cases, it serves as a protection layer for any physical damage, pathogens, protection against UV radiation, sensation, and regulation of temperature and moisture levels. Fish and humans share similar mechanisms of wound healing, including inflammation and tissue repair [19]. Furthermore, fish skin and human skin harbor a diverse array of pigment cells, known as chromatophores and melanocytes, respectively, which contribute to camouflage, UV protection, and color patterning. Pigmentation in both salmon and human skin is controlled by the distribution and production of pigments like melanin. Salmon can display a range of colors, from silvery-blue to deep red, due to the presence and arrangement of pigment-containing cells called chromatophores, similar to the melanocytes in human skin. [20]. On the other hand, there are dissimilarities such as salmon skin contains scale while this is absent in human skin.”

In the context of salmon skin, ultrasound and photoacoustic imaging presents an opportunity to explore its intricate layers, detect potential abnormalities, and monitor changes over time. These techniques can aid in assessing the impact of environmental factors, diseases, and stressors on the skin structure and function. Non-invasive, real-time imaging capabilities can contribute a deeper understanding of salmon skin morphology and its responses to various stimuli. Salmonid species, such as Atlantic and Pacific salmon, hold immense ecological and economic significance. Their unique life cycle, transitioning from freshwater to marine environments, places diverse physiological demands on their bodies, particularly their skin. The skin serves not only as a protective barrier against the external environment but also plays a crucial role in osmoregulation, local immune response, and sensory perception. Consequently, investigating the structural and functional aspects of salmon skin can offer valuable insights into their health, behavior, and overall well-being. Fish skin presents a complex heterogeneous medium where blood and pigment content is spatially distributed variably in depth [21]. Fish skin are composed of epidermis layer, dermis layer, and individual scales that form a textured surface [22]. Salmon skin contains a pigment called melanophores, xanthophores, and iridophores based on the skin location [23], and has an important role in social interaction, prey capture, and predator avoidance, all of which are crucial to their survival [24]. Morphological color changes in the skin occur due to variations in pigment concentrations and in the morphology, density, and distribution of chromatophores in three dimensions inside the skin [25]. PAM can image cells in 3-D which gives good spatial visualization and displays information with much accuracy. This can be proposed to detect diseases and infections, tumors and quality assessments by evaluating the thickness and texture of the skin. Fish skin can also act as a sensitive indicator of environmental change, where ultrasound and PAM can be used to access and monitor the changes in skin of fish population with shifts in the water quality, temperature, salinity and pollution. As these techniques are label-free, they takes advantage of the intrinsic optical absorption properties of endogenous chromophores [26] and difference in acoustic impedance for generation of contrast respectively.

Herein, we present label-free acoustic and photoacoustic imaging of salmon skin. To the best of our knowledge, this is the first demonstration of ultrasound and PAM as a method for the analysis of salmon skin in three-dimensions. Through a detailed analysis of the acquired images, this study seeks to elucidate the advantages and potential applications of this combined imaging technique. In the present study, PAM and ultrasound images are combined to visualize the three-dimensional (3D) structure of fish skin and its association with tissue structures in the skin layers. By overlaying the scanning acoustic image on top of the photoacoustic image, a more comprehensive and holistic view of salmon skin can be achieved. This visualization can help in identifying changes in the morphology, structure, or functional changes in the salmon skin resulting due to any infection or other parameters. In addition, PAM can be instrumental in the quantification of changes in multilayered morphology and endogenous components of fish skin.

## 2 Methods

### 2.1. (Ethics Statement)

The experiments were performed in compliance with the guidelines on animal research and approved by the Norwegian Food Safety Authority (FOTS ID28060), (Ethical committee- Ole Aamodt and Gunvor Knudsen (Case manager)).

### 2.2. (Sample preparation)

**Fig 1:**
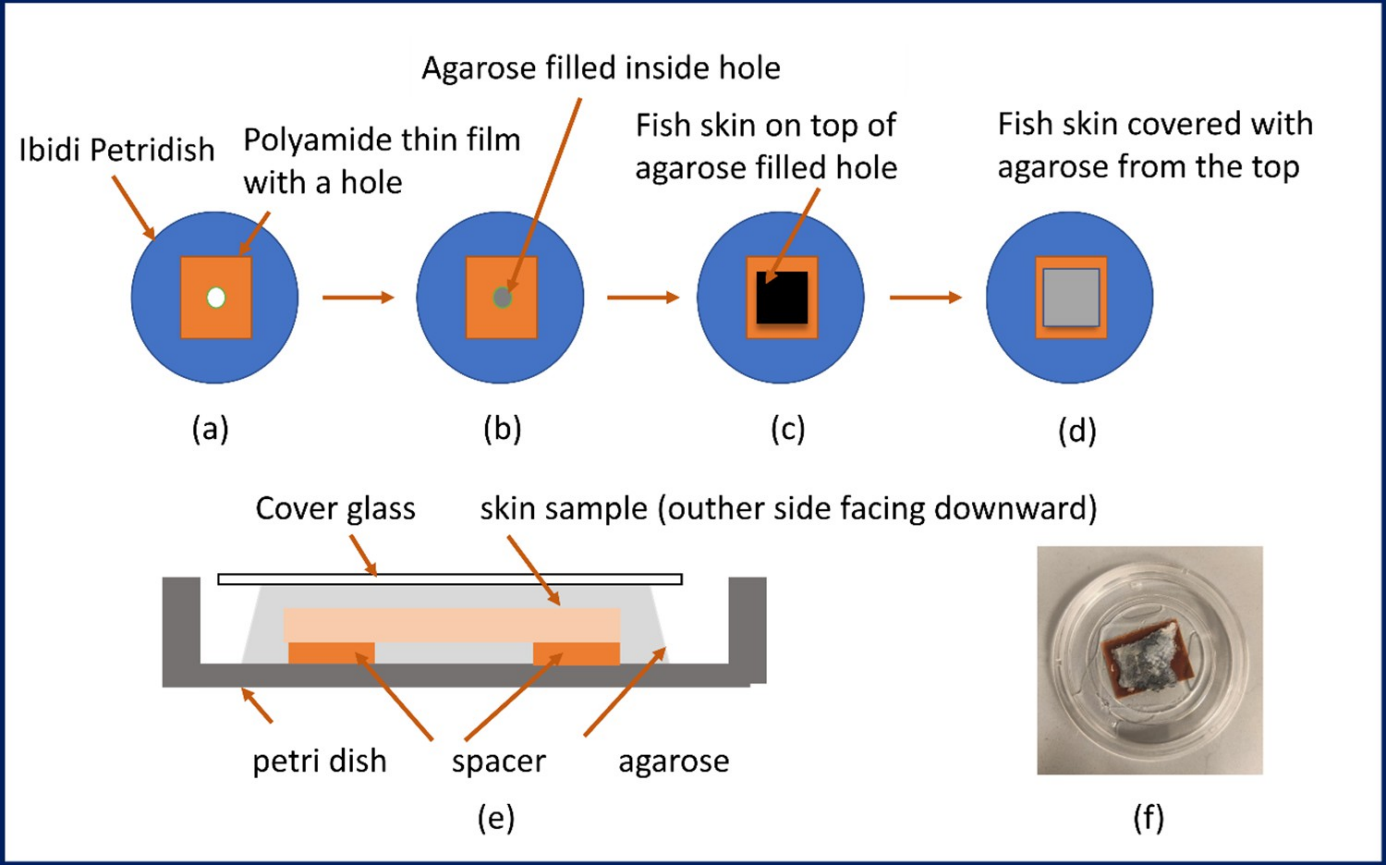
A cross sectional view of the petridish with the fish skin sample.

Fishes were kept and maintained in the Aquaculture Research Station, Kårvika, Tromsø. Fish skin near the middle of the dorsal fin was taken out using a sharp scalpel having an average weight of 175 ± 15 g. Removal of the muscle from the skin was done rendering a thin skin sample. The thickness of different fish skins varied from around 1.3 mm to 1.5 mm. A thin polyamide film of thickness 124 μm was pasted on the petridish to lift the sample, (Ibidi, 50mm, low) with a small hole of around 6 mm in it as shown in Fig 1(A), 1(B) and 1(C).

The optical properties of agarose such as the refractive index of agarose is close to water as agarose is a hydrogel containing a high percentage of water. At a concentration of 1%, agarose gels exhibit good optical transparency, allowing light to pass through with minimal scattering or absorption. The agarose has optical refractive index around (1.34–1.36) depending on the concentration and temperature which is also close to water (1.33) [27].

The speed of sound in agarose gel depends on the concentration of agarose and the temperature. Generally, the speed of sound in agarose gel is slightly higher than in water. For example, in 1% agarose gel which has been used in our case, at 20°C, the speed of sound is in the range of 1500–1550 m/s, which is higher than in water [28]. The acoustic impedance of water at 20°C is approximately 1.48 × 10^6 kg/(m^2^s). The acoustic impedance of agarose gel is generally higher than that of water due to its higher density. For a 1% agarose gel at 20°C, the acoustic impedance is around 1.58 × 10^6 kg/(m^2^s), which is higher than that of water [29]. The higher acoustic impedance of agarose gel compared to water can lead to stronger reflections at the interface between the two materials, potentially reducing the transmission of photoacoustic or ultrasound signals.

The ultra-low temperature agarose (Sigma-Aldrich) was prepared by putting 0.15 grams in 10 mL of distilled water and heating it with a magnetic stirrer to its melting temperature. The agarose was cooled for some time before it was poured on the skin sample. A small drop of agarose was poured into the circular hole and then left to solidify. The thin skin sample was then kept on a cover glass and 1.5% of agarose was poured onto the skin sample. The cover glass having the skin sample as shown in Fig 1(D) covered with liquid agarose was put on the petridish and pressed slightly. Later, the glass cover was taken out gently and an embedded fish skin sample sandwiched between two agarose layers was formed. The petridish skin sample was poured with deionized water for better acoustic coupling. The sample was preserved in an incubation chamber at 4° C before imaging The fish skin samples were further processed and imaged the same day or the next day. Fig 1(E) shows the cross-section view of the petridish having the polyamide film with a hole and the salmon skin on top of it and Fig 1(F) illustrates the optical image of the skin sample.

### 2.3. (Experimental setup)

**Fig 2:**
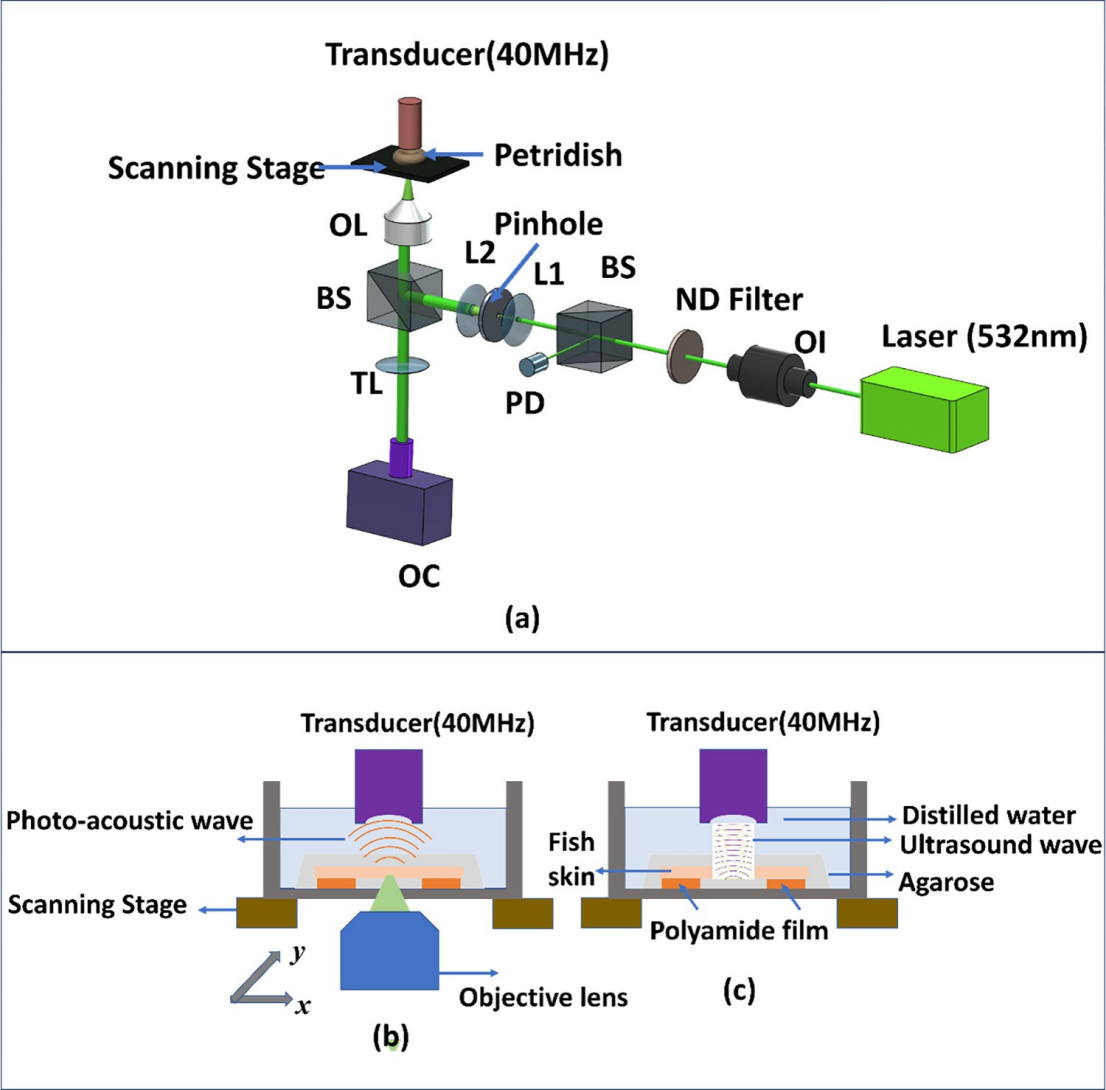
(a) A simplified PAM experimental setup. OI: Optical isolator, BS: Beam splitter, ND filter: Neutral density filter, PD: Photodiode, OL: Objective lens. TL: Tube lens. (b) The schematic view of the PAM setup (c) A simplified experimental setup for SAM.

In order to investigate the photoacoustic response from the fish skin, an experimental setup was designed around an inverted microscope (Leica DMi8) in transmission mode with additional optical and acoustic components as shown in Fig 2(A). A high-precision scanning stage (ASI MS-2000) was used on the microscope to utilize photoacoustic images of the fish skin samples using mechanical raster scanning. A customized LabVIEW program implemented the control of stages. This program also assured synchronization with the optical and acoustic components, ensuring the needed communication and data transfer with the time-critical digitizing implemented in FPGA hardware from National Instruments. An optical camera was also used in wide-field reflection mode to provide optical images of the investigated samples with 4X magnification for a large field of view for better overlapping of PAM images.

The entire set-up was built on an optical table to minimize the effects of mechanical vibrations. The sample was irradiated with 532 nm, Q-switched, mode-locked pulsed laser (Elforlight SPOT-20-200-532) that yields pulses with widths down to 1.6 ns, repetition rates up to 10 kHz, and energies up to 20 μJ. An optical isolator was placed immediately after the laser to avoid unwanted back reflection into the laser cavity and allow one-way transmission of laser light. The laser pulse was also spatially filtered using a 50-μm diameter pinhole to reduce different aberrations, and unwanted spatial variations and create a uniform Gaussian intensity distribution. After filtering, the beam was brought into the inverted microscope through an infinity port and focussed by a Leica objective lens (depth of field: 10μm, magnification- 10X). The theoretical lateral resolution for the system could be determined using the formula given in Eq (1) where The objective lens used in the experiment had a numerical aperture (NA) of 0.3, wavelength (λ) 532 nm [30].


$$\mathrm{Lateral}\;\mathrm{Resolution}=0.51*\frac{\lambda }{NA}=0.904\;um.$$ (1)

The acoustic parts of the setup, includes a focused ultrasound transducer (Olympus, Serial number- 200637)) with specified centre frequency (40 MHz) and f-number (2). In the ultrasound imaging as shown in 2(c) the transducer sends the ultrasound beam and receives the reflected acoustic waves while in PAM it acted only as a receiver for the photoacoustic waves. The transducer is then mechanically raster scanned over the surface of the sample. As it moves, it emits ultrasonic waves into the material. The sample inside the petridish was placed on the scanning platform with distilled water to lower the attenuation for acoustic waves and better acoustic coupling with transducer in focus. The theoretical axial resolution of our PAM system can be calculated using the formula given in Eq (2) where The velocity of sound in water is approximately Vs  =  1500 m/s, the bandwidth (f) of the ultrasonic transducer is 40 MHz. [30]

$$\mathrm{Axial}\;\mathrm{Resolution}=0.88*\frac{Vs}{f}=33\;um.$$ (2)

The ultrasound transducer and the laser were coaxially and confocally aligned before imaging for high sensitivity and to obtain overlapping of the optical and acoustic focus.as illustrated in Fig 2(B). Subsequently, the acoustic signal from the transducer were amplified and low-pass filtered with a customized pre-amplifier, and then digitized with and a high-speed 12-bit digitizer (NI-5772) to obtain good signal integrity. The acoustic waves were detected 32 times and averaged to reduce noise in each measurement. The laser fluence was kept as 0.00295 mJ/cm^2^.which is within the limit of ANSI standards [31].

**Fig 3:**
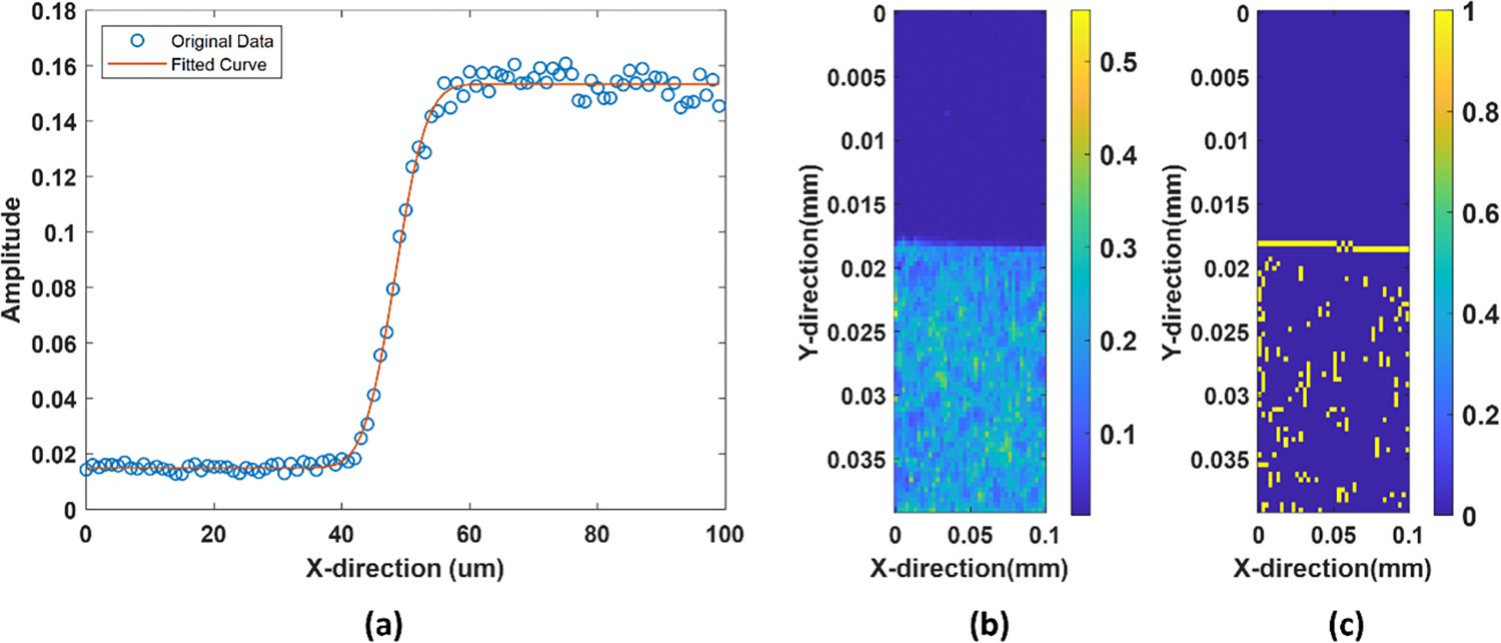
(a)line profile of a sharp object fitted with error function (in red) (b) Photoacoustic image of the edge (c) Photoacoustic image showing the boundary.

The experimental measurement of the lateral resolution was done using an edge sharped structure with a clear non absorbing and absorbing layer with a transition at the edge. The photoacoustic signal profile was analyzed across a sharp edge or boundary within the imaged sample and fitted to an appropriate edge function to the measured data as shown in Fig 3(A). In our case, This was fitted with a gauss error function in MATLAB. From the fitted error function, the characteristic width or spread parameter that describes the transition region across the edge was determined. This parameter is typically denoted as σ (sigma) for the Gaussian error function. The lateral resolution is defined as the full width at half maximum (FWHM), which is related to σ by FWHM = 2.355σ. The obtained sigma value ranges from 4.25 to 4.4. The lateral resolution for our system is 9–10 μm. Fig 3(B) shows the image of the edge while Fig 3(C) shows the image with edge detection mechanism.

The axial resolution in photoacoustic imaging is typically related to the temporal characteristics of the detected photoacoustic signal. Specifically, the axial resolution is determined by the width of the photoacoustic wave packet propagating in the direction perpendicular to the imaging plane. The full width at half-maximum (FWHM) of the Hilbert transform of the photoacoustic signal can be used to estimate the axial resolution of the photoacoustic imaging system, as it is directly related to the width of the photoacoustic wave packet in the axial direction. The experimental axial resolution was found out to be 33–35 μm.

## 3. Results and discussions

The experimental data stored in the H5 (Hierarchical Data Format) format was analyzed using a customized software program in MATLAB R2021a. The acoustic signals were sampled at a sampling frequency at 400 MHz. The bias value for the signal was subtracted for calibration. The trigger value for the SAM and PAM signals were adjusted accordingly in time in order to make them equal size. This was done because time taken by the acoustic waves to reach the transducer is almost twice to that of photoacoustic signals. A selected window was chosen such that the signal coming from the fish skin is taken out and the noisy part is removed. A specific customized colormap was chosen for the overlayed image. Customized colormaps enable the assignment of various color schemes or gradients to each modality, allowing for easy separation between SAM and PAM information within the overlay image. We present an overlay of the SAM and PAM images, with each modality represented by a custom colormap. For example, in the image 5-6(a) and (b), the PAM is represented by hot, and SAM is represented by grayscale. For the SAM image, a customized colormap is designed to represent the ultrasound data using shades of a specific color. This colormap can be tailored to highlight specific features or intensities within the SAM data, helping to emphasize areas of interest or reveal structural details.

One of the key advantages of colormaps is their ability to enhance the visual representation of data. By assigning distinct colors to different data ranges or levels, colormaps make it easier to distinguish patterns, contrasts, and features that might be difficult to discern from a grayscale intensity map. This visual encoding assists in highlighting subtle variations and gradients within the data, providing a more comprehensive and accurate interpretation. A direct intensity projection map provides only a 2D representation of the combined data, the use of customized colormaps can convey additional information by encoding different modalities or properties into different color channels. This effectively adds an extra dimension to the visualization, allowing for a more comprehensive representation of the multimodal data. In contrast, a direct intensity projection map with an envelope provides a more straightforward representation of the data, displaying variations in intensity or brightness.

This was done by creating a customized matrix for colormap where first half part the matrix was allotted with gray colormap, and the other half was assigned with inverted colormap. The values were interpolated using the piece wise function, which generates a linear gradient. This function constructs a cubic Hermite spline with continuous first derivatives and chooses the coefficients to ensure that the curve passes through the given reference points, this method generates a custom colormap that gradually changes from one colormap to another using the interpolation values. The interpolated function was made to monotonically increase between the breakpoints. This can be useful for visualizing data with varying intensity levels. A customized alpha map which controls the transparency of the image was also chosen using piecewise cubic Hermite polynomial interpolation analogous to colormap. Unlike some other interpolation methods, cubic Hermite interpolation considers both the function’s values at data points and its derivatives at those points, allowing for a more flexible and accurate interpolation. This technique is especially useful when you have data points with associated values and derivatives (or slopes) at those points. The goal is to construct a smooth curve that passes through the given data points while maintaining continuity in both function values and derivatives. The three-dimensional matrices responsible for the SAM and PAM data were made equal in size then combined by multiplication. This is because the acoustic signal in z-direction were made equal from the trigger position. The starting point of PAM data relative to SAM data, considering trigger positions was adjusted. Absolute time vectors for SAM and PAM were adjusted by a trigger delay. In order to make both the signals equal, the time vector responsible for photoacoustic signal was halved. This is because the acoustic signal takes twice the time as photoacoustic signal. In this way, the matrices responsible for SAM and PAM were made equal in size temporally and spatially.

The specific colormaps and alpha values can be adjusted to achieve the desired visual representation of the data, allowing for a combination of color and transparency in the visualization with varying levels of opacity.

**Fig 4:**
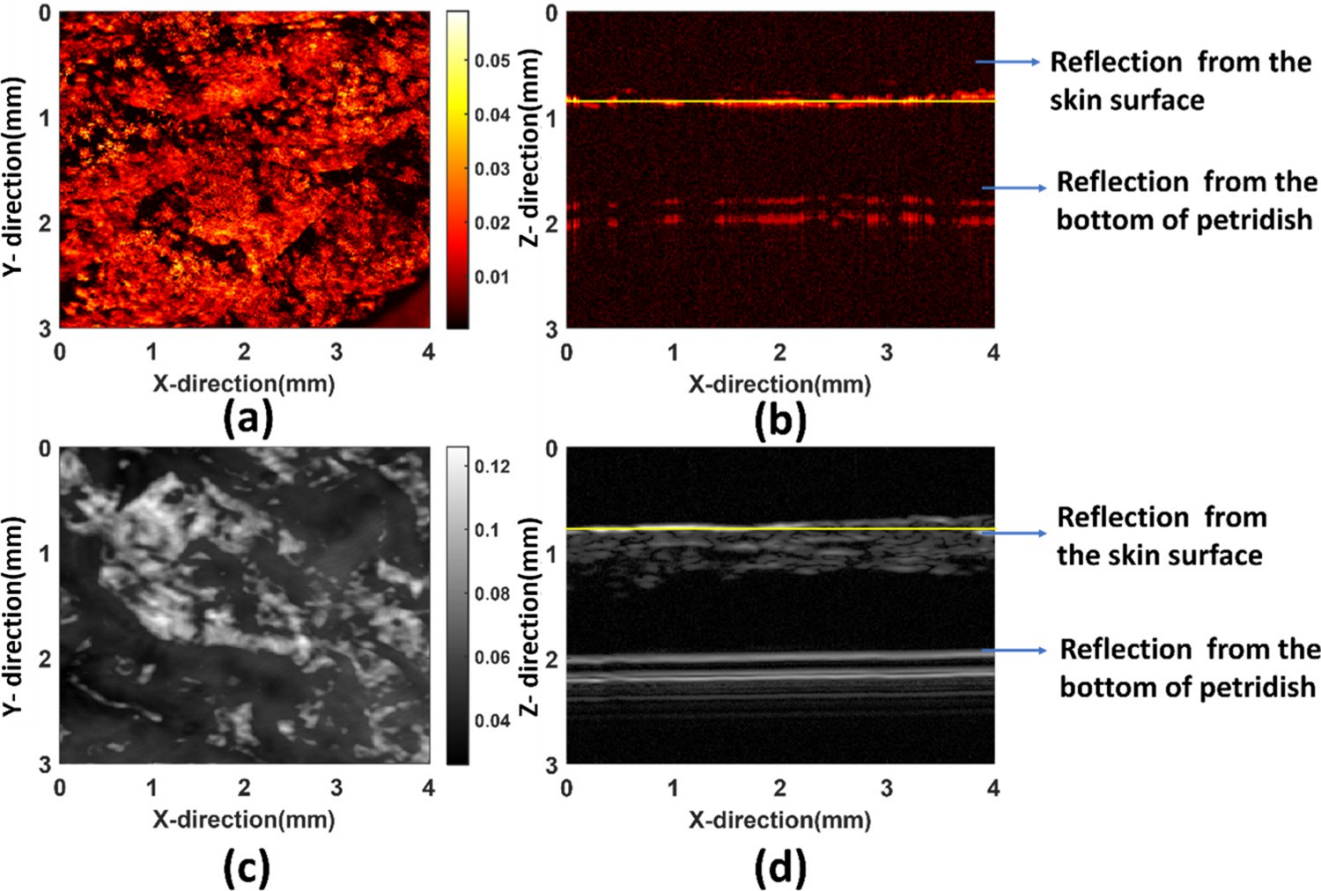
(a)C-scan for PAM (b)B-scan for PAM, Hot colormap is chosen for PAM representation (c) C-scan for SAM (d) B-scan for SAM, gray colormap is chosen for SAM representation. The yellow line in B-scan indicates the region for which C-scan has been shown. The size of the C-scan images is 4mm*3mm and the pixel size is 10 μm.

Fig 4(A)shows the C-scan image obtained by maximum amplitude projection, a visualization technique which is used to display the maximum amplitude or intensity values obtained from the photoacoustic signals generated where the pixel value at each point in the image corresponds to the maximum amplitude (or intensity) of the signal within a given depth or thickness of tissue. The layer responsible for the absorption for PAM image generation in Fig 4(B)is thinner while in the case of ultrasound image Fig 4(D) it is much thicker, so there are reflections coming from bottom of the petridish in case of SAM image. Fig 4(C) reveals the maximum projection image for SAM where the structural morphology of the skin surface can be observed. Fig 4(C) provides a representation of the acoustic mismatch between different tissues. In the SAM image, the mechanical and the elastic property of the tissue plays a significant role for generating the image contrast. The PAM image represents the initial pressure distribution produced by the deposition of optical energy thus making the contrast based on absorption. The mean ratio of the photoacoustic amplitudes between the optical absorbers and the background is approximately 14:1, which demonstrates a high endogenous optical-absorption-based contrast. It can be observed that the scattering is also coming from the deeper parts of the skin unlike PAM. It can be observed that a greater tissue differentiation and specificity is possible with PAM image than SAM image. It is because the difference in absorption between tissues can be much larger than the difference in the acoustic impedance. From the Fig 4(B) and 4(D)it can be observed that the scattering is coming from the top layer of the skin or close to the skin surface and then slightly weaker signal from the bottom of the petridish. The sample preparation was done in such a manner that we can differentiate the signal coming from the top and bottom.

**Fig 5:**
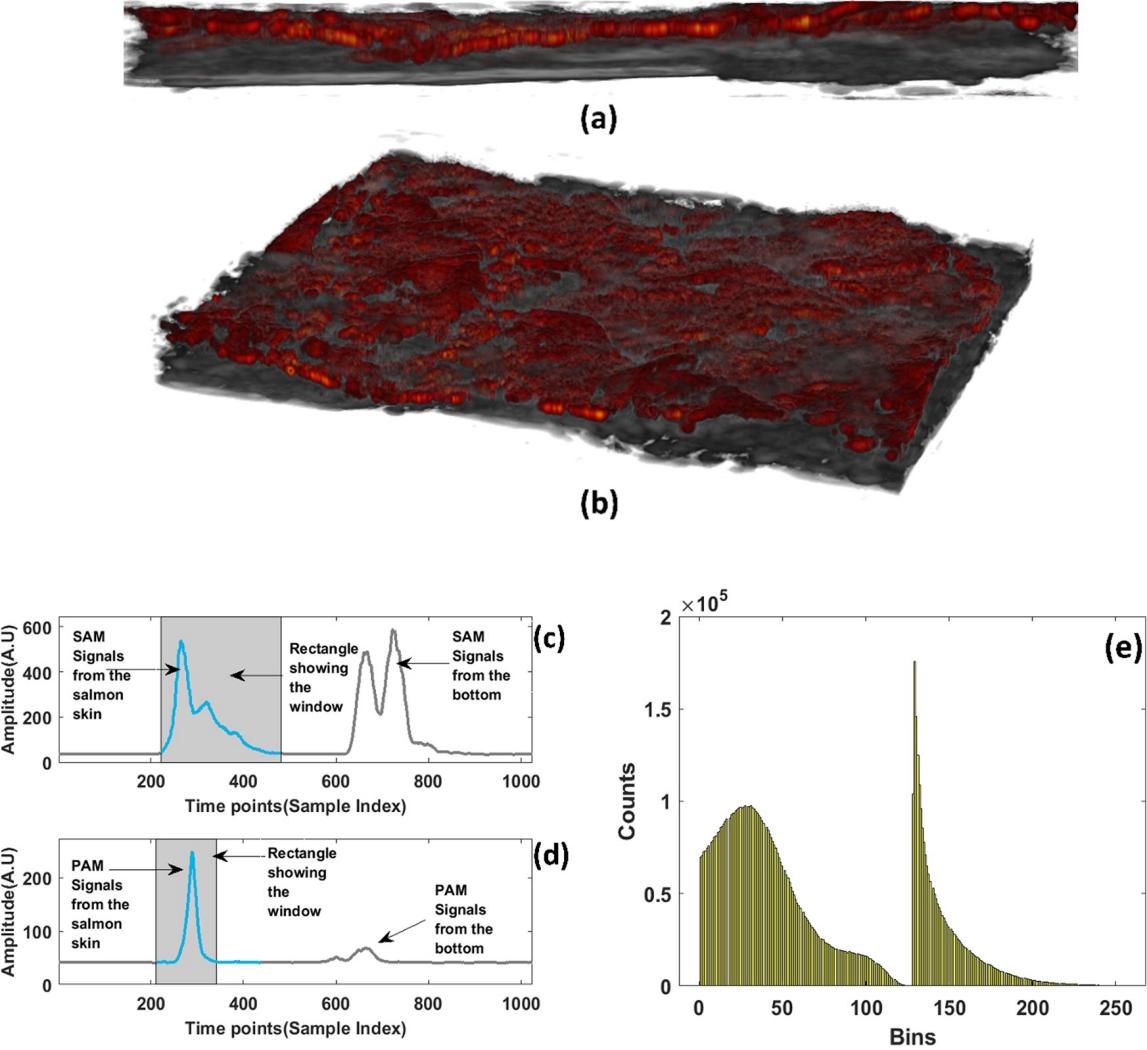
(a) side view of SAM & PAM overlayed image from dataset1 having dimensions (4mm*3mm) (b) angular view representation of the overlayed image(c) The SAM signal where the white shadowed part shows the signal responsible for the image. (d) The PAM signal where the white shadowed part shows the signal responsible for the image and the amplitude (Arbitrary units) (e) Volume histogram of the signal where the bins are from 0 to 256.

**Fig 6:**
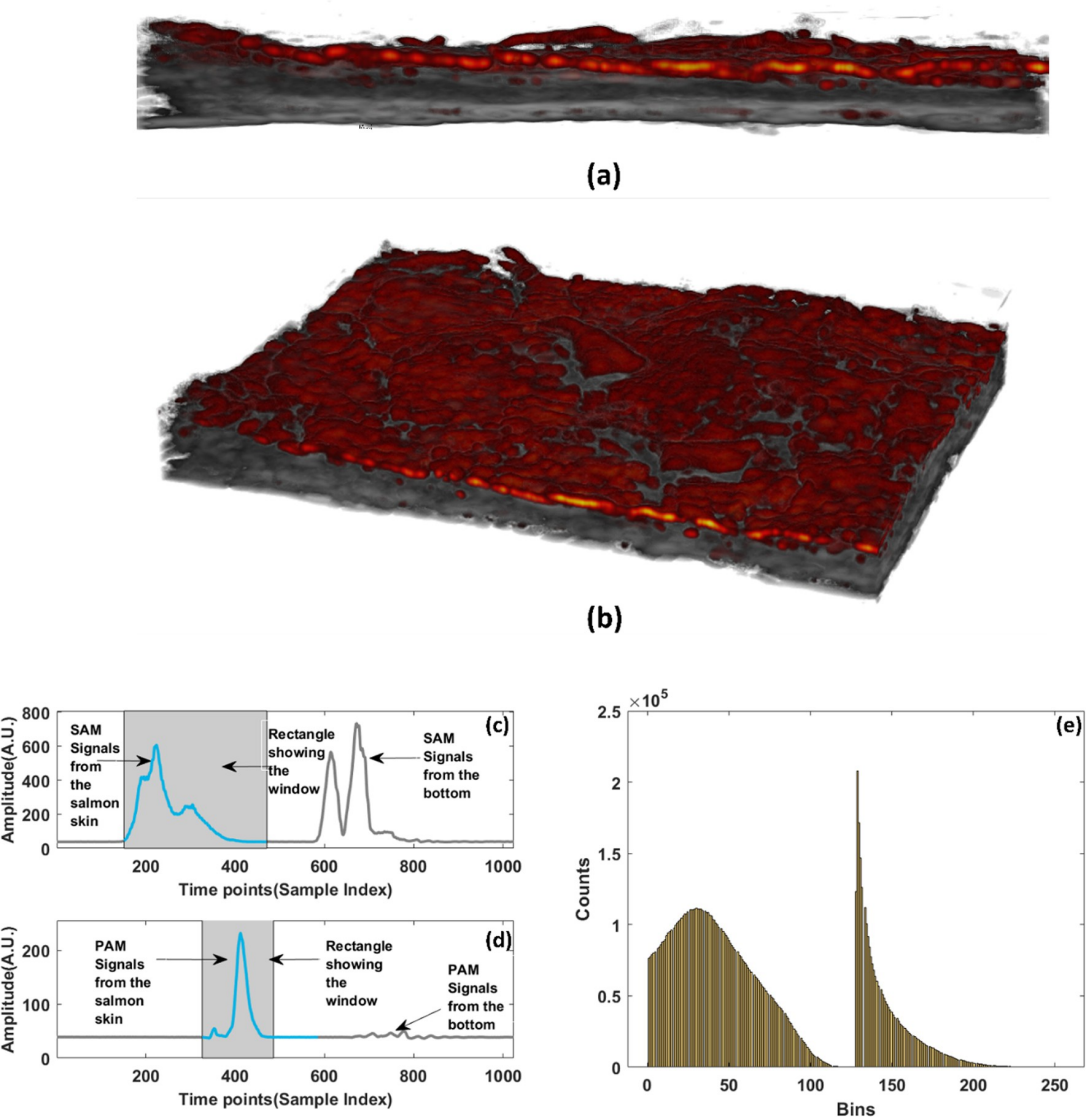
(a) side view of SAM & PAM overlayed image from dataset2 having dimensions (4mm*3mm) (b) angular view representation of the overlayed image (c) The SAM signal where the white shadowed part shows the signal responsible for the image (d) The PAM signal where the white shadowed part shows the signal responsible for the image and the amplitude is in Arbitrary units(d) Volume histogram of the signal.

The major optical absorbers in the visible spectral range for salmon skin tissue include carotenoids such as melanin [32]. the primary optical function of melanin is absorption, and that Rayleigh scattering in melanin is negligible [33]. Changes in color, or total loss of color, notifies that there has been a decomposition or structural modification of the carotenoid. This, in turn, can be related to the camouflage, response to external stimuli and communication among individual fishes [34].The intensity of the color provides the basis for a quantitative determination of carotenoids. Consequently, PAM can act as a sensitive detector for the pigments with high contrast and specificity, is suitable for imaging the volumetric morphology of subcutaneous microvasculature in vivo. Photoacoustic imaging gives a time series to be analyzed together with time series corresponding to every pixel, which makes it advantageous over any other purely optical imaging modality. In this paper, we have presented two datasets for two different healthy salmon samples. We have taken several measurements for consistency out of which we are representing the best results. Figs 5(C) and 6(C) shows the ultrasound signals and 5(c) and 6(c) below demonstrates the PA signal coming from the salmon skin. “In the Figs 5(B) SAM and 6(C) PAM signals are represented respectively. This representation is done after taking the Hilbert transform of the signal which gives an envelope of the signal. The shadowed part reveals the window of signal responsible for the image in both (b) and (c). In the 4(b) we can see the two signals where the first one is corresponding to the signal from fish skin while the second signal is due to the strong reflection coming from the bottom of the petridish. In the 4(c), the first signal is the photoacoustic signal coming from the salmon skin sample while the second one is a weak reflection from the bottom of the petridish.”

These signals are also shown for the same fish sample from the same area and the pixel generating the maximum signal is shown. The shaded grey area of the signals in Figs 5(C), 5(D), 6(C) and 6(D) shows the signals taken out for generating the overlayed image. Using the pulse-echo ultrasound method, the speed of the sound inside the salmon skin was calculated at around 1550 m/s for the thickness of the salmon skin as 1.3 mm. From the time series in the first PA signal has a peak coming from the top mainly and then the next signal is coming from the petridish. In the case of the SAM signal, apart from the signals from the top of the skin and the bottom of the petridish, the attenuated scattering also comes from the middle sections which consists of scales and a part of muscles too. Figs 5(E) and 6(E) shows the volume histogram of the signal showing the frequency distribution of values within the signal dataset. The larger peak likely corresponds to the ultrasound matrix data from the salmon skin tissue. Ultrasound is generally good at visualizing structural details and boundaries within biological tissues like fish skin. The smaller secondary peak represents the photoacoustic matrix data. Photoacoustics can provide functional information by detecting optical absorption contrasts, which could be related to the presence of specific chromophores or pigments in the salmon skin. The bimodal nature arises because ultrasound and photoacoustic signals have different physical origins—acoustic waves versus optical absorption and thermoelastic expansion, respectively. Hence, their value distributions are distinct but complementary.

**Fig 7:**
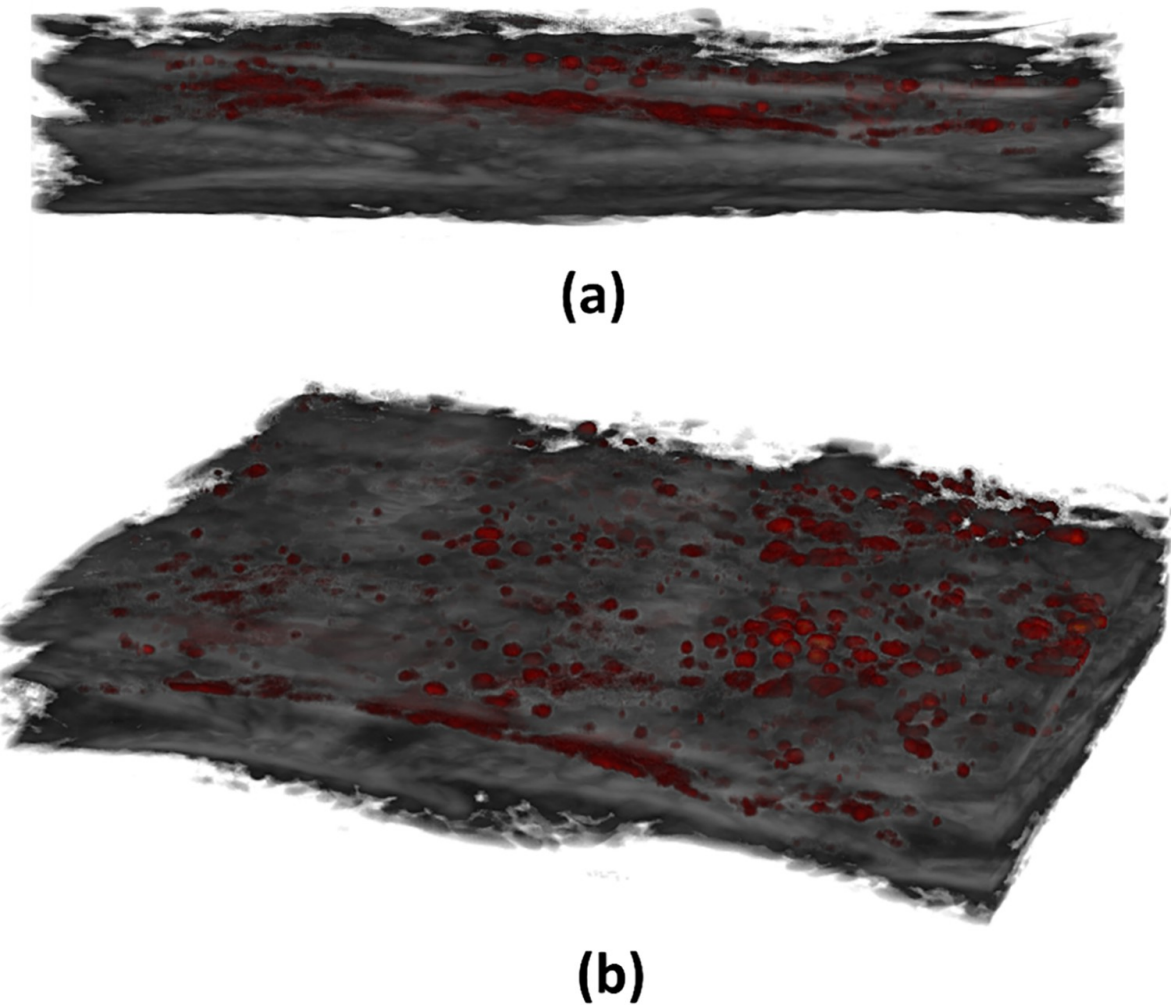
(a) side view of SAM & PAM overlayed image from dataset3 which has scattered pigments having dimensions (4mm*3mm) (b) angular view representation of the overlayed image.

We show data from two different salmon skin sample in this article for demonstrating the consistency of our results. In the Figs 6(A) and 7(A), we can see the pigment patterns present on the salmon skin surface and beneath the surface. In the image we can see individual fish cells and the cluster of cells. Overlaying the images on top of each other allows for a direct comparison and correlation of features observed in both modalities. It can be analyzed that there are scales present beneath the skin which can contribute to the scattering. The pigment cells seem to follow the scale-tissue curvatures quite closely and the distribution of pigment is non-uniform. The optical absorption of the salmon skin has a significant impact on the photoacoustic signal’s amplitude. In SAM, there are many scatterers of different sizes coming from a thick layer leading corresponding to different signals while in PAM, the signals are mainly corresponding to the top or close to top layer of the skin since the primary chromophores responsible for light absorption would be the pigments present in the top layer of the skin or close to the top layer.

Fig 7 shows a fish skin with slightly high thickness and pigment distribution which is non-uniform and scattered. The purpose of showing the dataset (3) is to present a case of fish where the pigments are less than other datasets. Based on three datasets, we see different pigment patterns in three different salmon fish. This is an indication that fish skin pigments vary based on several external factors and are different.

**Fig 8:**
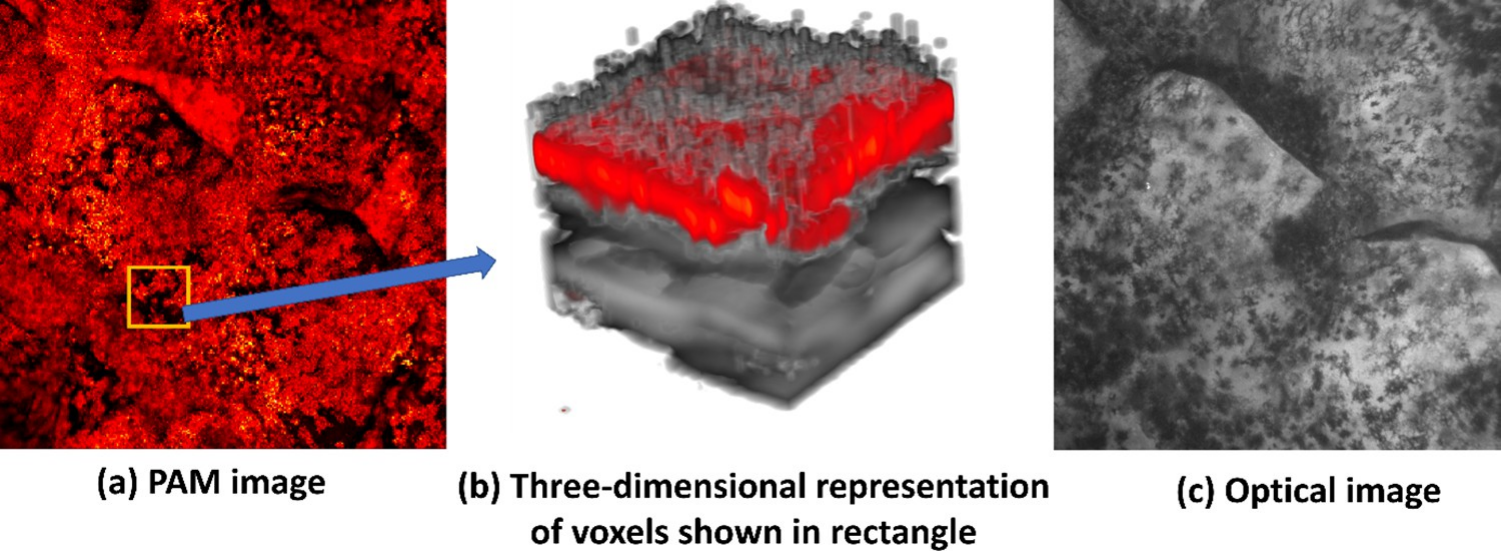
(a) PAM image(4mm*3mm) showing the optical absorption map. (b) A rectangle showing the area taken for three-dimensional voxel representation in (c) The optical image of the same region (The optical image was taken with 4X lens for high field of view and better overlapping).

The PAM data seems to be strongly correlated with optical images of the skin as can be seen in Fig 8(A) and 8(C). However, PAM typically includes more spots/features which are not visible in the optical images as shown in the Figure. This is due to scatterers present not only on the surface but throughout the volume of the skin as shown in Fig 8(B). It also shows that the skin is a bit tilted, and the scatterers are highly dense. It can be evidently observed that the pigments are not only present on the skin surface but also present beneath the skin. Here, we can see that photoacoustic image has an advantage over optical images as it can provide us to see features at much deeper level than just surface imaging with optical imaging.

**Fig 9:**
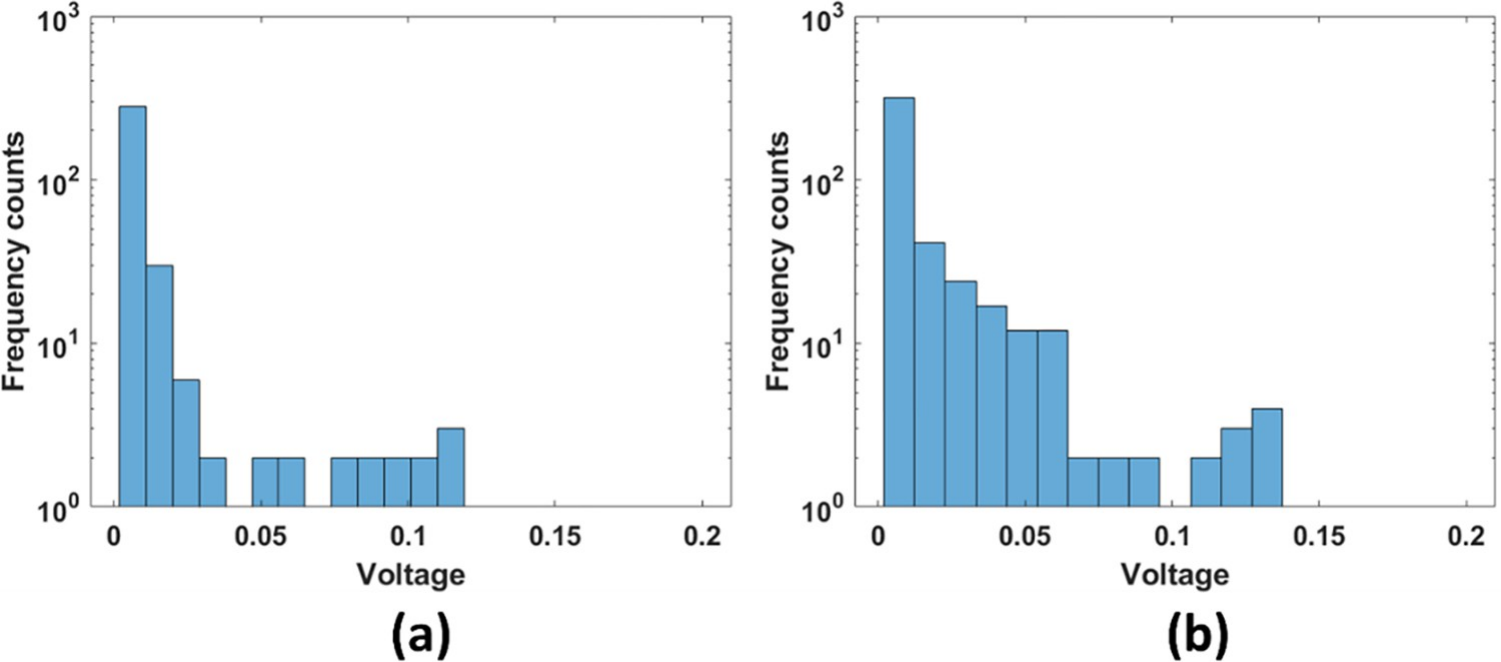
Histogram for calculation of voxels which contribute to the signal (a)PAM (b) SAM signal. The unit of voltage is in Volts.

The presented histograms in Fig 9(A) and 9(B) shows a typical amplitude distribution for the received PAM and SAM signals. Both the PAM and SAM histograms show that voxels producing small signal amplitudes are occurring most frequently. For PAM it is believed that individual pigment cell having typical sizes between 10 to 100 um [35](depending on which part of the fish it is taken from) can be resolved in the lateral direction due to a significant smaller laser width (around 5 μm in our case). It is therefore believed that the smallest and most likely PAM scatters occur from voxels contain only one pigment cell. Larger PAM amplitudes will occur from having several pigment cells within the voxel volume, or from single cells yielding higher absorption. However, since the PAM histogram does not show any statistically significant peaks at some scatter strength as would be expected for pigments in clusters or patterns, it suggests a rather homogonous pigment distribution. The histogram for the SAM amplitudes shows a statistical significant peak at a specific amplitude. This peak is believed to be due to the binary scattering properties of the fish skin, with fish scales yielding a quite high acoustic impedance and the surround tissue having a much lower impedance.

The overall shape of the histogram, with a tall primary peak and a few smaller secondary peaks, indicates a relatively heterogeneous tissue structure within the salmon skin. The variations in voltage amplitudes correspond to differences in acoustic impedance and absorptions differences across different tissues in SAM and PAM respectively. It can be observed clearly that the small scatterers are present in larger amount, the big scatterers are small in quantity both in SAM and PAM images. A minimum threshold is defined and above which all the voxels are counted and summed up. that exceed a certain voltage threshold and compute the average value of these voxels relative to the total number of voxels. By looking at the percentage of voxels contributing to the signal, it was found that for PAM contributed to 0.069%, SAM contributed to 0.22%.

In summary, this study presents a comprehensive investigation into the application of 3D acoustic and photoacoustic microscopy for the analysis of salmon skin. PAM could non-invasively access the multilayered morphological skin structure of salmon fish for high resolution imaging and analysis without the need of labels. We have shown that compared with conventional optical detection methods, an approach of representation by overlaying the fish skin images on top of each other obtained via SAM and PAM gives more deeper look. This method of visualization enables an insight on distribution, variation, and composition of pigments inside the live salmon skin without any tissue related processing and fixing. The scanning acoustic image provides structural information, while the photoacoustic image offers functional information. The unique visualization of photoacoustic and scanning acoustic images has applications in various fields, such as biomedical research and clinical practice. It can be used in areas like tumor imaging, vascular imaging, functional brain mapping. Our findings hold significant implications for the aquaculture industry, offering a non-destructive and efficient means of monitoring the health and well-being of salmon populations via skin. The ability to visualize skin structures and vascular patterns could facilitate early detection of any type of stress and infection leading to any disease, ensuring prompt intervention and management.

However, it is important to acknowledge the limitations of the current study. Further refinements in imaging technology and data processing methods are necessary to optimize the resolution, penetration depth, and image quality. In order to improve the axial resolution of the PAM system, higher frequency ultrasonic transducers can be used to obtain the thickness of each skin structure layer more accurately. Finally, the current imaging requires approximately 10 min to cover an area of 4 mm × 3 mm. In future, a faster scanner, such as a voice-coil scanner or a micro-electro-mechanical-system-based scanner, would be beneficial in eliminating the effects of motion and improving clinical evaluation. Each fish we picked had some uncertainty in terms of their difference of size, weight, same distance from the fins.

Further studies can be done in future using this method to understand the evolution of pigments, the mechanism of camouflage, disruptive coloration etc. and potential adaptations to different environments. Further research can also tell us about the concentration and density of pigments using photoacoustic microscopy. There is so much still to learn about the composition, expression, and distribution of pigment-based color patterns, its relation to stress, mating and communication, health condition in salmon fish. Absolute quantification of these functional parameters will allow for the correct determination of the physiological status of tissue, pigment quantification and accurate diagnosis of pathological conditions, effect of environmental factors. Studies can be conducted to evaluate the stiffness of the salmon skin using ultrasound which can be used as a sign of health and structural integrity. Optical attenuation coefficient inside the skin can be measured depending on the wavelength of laser, skin pigments, and the skin’s absorption and scattering characteristics.
